# Selectivity of Relative Humidity Using a CP Based on S-Block Metal Ions

**DOI:** 10.3390/s22041664

**Published:** 2022-02-21

**Authors:** Amalia García-García, Víctor Toral, José F. Quílez del Moral, Alberto Galisteo Pretel, Diego P. Morales, Alfonso Salinas-Castillo, Javier Cepeda, Duane Choquesillo-Lazarte, Marco Bobinger, José F. Salmerón, Almudena Rivadeneyra, Antonio Rodríguez-Diéguez

**Affiliations:** 1 Department of Inorganic Chemistry, Faculty of Science, University of Granada, 18071 Granada, Spain; amaliagarcia@correo.ugr.es; 2 Pervasive Electronics Advanced Research Laboratory (PEARL), Department Electronics and Computer Technology, University of Granada, 18071 Granada, Spain; vtoral@ugr.es (V.T.); diegopm@ugr.es (D.P.M.); jfsalmeron@ugr.es (J.F.S.); 3 Department of Organic Chemistry, Faculty of Science, University of Granada, 18071 Granada, Spain; jfquilez@ugr.es (J.F.Q.d.M.); albertogapre@ugr.es (A.G.P.); 4 Department of Analytical Chemistry, Faculty of Science, University of Granada, 18071 Granada, Spain; alfonsos@ugr.es; 5 Department of Applied Chemistry, Faculty of Chemistry, University of the Basque Country (UPV/EHU), 20018 Donostia-San Sebastián, Spain; javier.cepeda@ehu.eus; 6 Laboratorio de Estudios Cristalográficos, IACT, CSIC-Universidad de Granada, Avda. de las Palmeras 4, Armilla, 18100 Granada, Spain; duane.choquesillo@csic.es; 7 Institute for Nanoelectronics, Technical University of Munich, 80333 Munich, Germany; marco.bobinger@tum.de

**Keywords:** flexible substrate, spray deposition, moisture content, interdigitated electrodes, screen printing, sodium, perylene

## Abstract

Herein, we present the syntheses of a novel coordination polymer (CP) based on the perylene-3,4,9,10-tetracarboxylate (pery) linkers and sodium metal ions. We have chosen sodium metal center with the aim of surmising the effect that the modification of the metal ion may have on the relative humidity (RH) experimental measurements of the material. We confirm the role of the ions in the functionalization of the deposited layer by modifying their selectivity towards moisture content, paving the way to the generation of sensitive and selective chemical sensors.

## 1. Introduction

Water vapor is present in the air and is the most varied component. At the same time, atmospheric conditions are essential for many processes of our daily life, especially in an industry environment [1]. For example, electronics or food industry need this type of control during fabrication and transportation. Due to this fact, the sensing of relative humidity (RH) with integrated and cost-effective solutions is of great interest for broad types of industries.

RH sensors are typically based on ceramic materials, such as aluminum oxide, semiconducting materials, such as SiO_2_, or polymers [1,2]. Additionally, 2D materials have appeared as a solution to flexible and highly integrable sensors such as, for example, carbon nanotubes [3] or silicon nanosheets [4]. More recently, coordination polymers (CPs), including metal–organic frameworks (MOFs), are a relatively new class of materials that have attracted great interest due to their structural and topological diversity, as well as the properties that arise from their structural features [5,6,7,8]. This class of materials has also been used for RH sensors, as shown in [9], where a MOF is used to create a luminescent and impedance spectroscopy sensor. In [10], a luminescent RH based on MOF polymer is presented.

These sensors are typically based on a variation of capacitance or resistance of the materials, i.e., they are passive sensors. Active devices that react to RH variations are not iso common and are normally oriented to mechanical systems [11]. In the case of electronics devices, several works exist on RH sensing based on field effect transistor (FET) [12,13]. Additionally, organic field effect transistors (OFETs) used as humidity sensor can be found in the literature [14,15,16,17]. However, these devices are mainly based on the variation of threshold voltage or conductivity of the channel, so they cannot be considered an active device. MOFs have been demonstrated to open the possibility of an electronic humidity actuator [18,19]. In previous works, the use of K-Pery MOF to create a humidity actuator was uncovered [20]. However, it was not studied if the response of K-Pery was due to the response of the perylene or the metal center employed.

For this reason, we decided to study this point, demonstrating the possibility of modifying perylene-based MOF to react to humidity by using different metal centers. In this work, we present a new CP based on the perylene derivative (pery) ligand and sodium metal ions to investigate the possibilities to control the selectivity to RH of this material. Sodium was used to modulate the metal–organic architecture of the K-Pery material due to the smaller ion size. Furthermore, spray coating was employed as a more repeatable method to preserve the material, and the temperature dependence was also analyzed. Spray coating is also a scalable fabrication method, which brings the possibility of easily translating these devices for mass production. The rest of the paper continues with materials and methods in Section 2. Then, in Section 3, the results are presented. Section 4 continues with the discussion of the results. Finally, conclusions derived from this work are exposed in Section 5.

## 2. Materials and Methods

### 2.1. Synthesis of Sodium-Based Metal–Organic Framework

**Figure 1:**
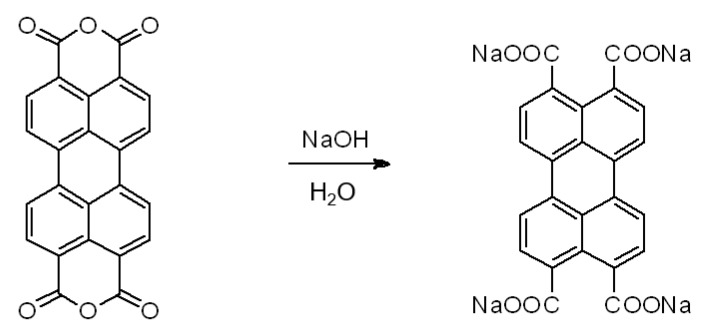
Synthesis of Na-Pery.

To a solution of perylene dianhydride (392 mg, 1 mmol) in 20 mL of distilled water, a solution of 166 mg (4 mmol) of NaOH in 10 mL of distilled water (Figure 1) was added. The mixture reaction was stirred at room temperature for 15 min. After this time, the color of the solution, initially dark red, turned to orange. This green solution was UV active. Solvent was then removed under infrared light for 24 h to afford a 96% yield (498 mg, 0.96 mmol) of micro-crystalline powders of Na-Pery.

The resulting solid was studied by elemental analysis (ELEMENTAR VARIO EL III, equipped with a thermal conductivity detector) combined with inductively coupled plasma (ICP-AES, carried out on a Horiba Yobin Yvon Activa spectrometer), from which the content of the solid could be estimated. The obtained results are concordant with the C_48_H_80_Na_5_O_47_ formula (Calcd.: C, 37.83; H, 5.29; Na, 7.54. Found: C, 38.05; H, 4.92; Na, 7.68), confirming the purity of the sample (see ESI for further information).

### 2.2. Preparation of the Ink and Spray Coating

The tested sensing material, Na-Pery, was dissolved in deionized (DI) water in a weight content of 0.6 wt%, respectively. In order to dissolve the material, a bath sonication treatment for a duration of 5 min was applied (Bransonic^®^ Ultrasonic Baths). The as-prepared solution was used as inks and sprayed onto polyimide (KaptonHN) substrate from DuPont^TM^, employing a handheld airbrush Triplex II from Gabbert (Leipzig, Germany). The sample was attached to a hot plate Rt2 from Thermofisher Scientific^TM^ at a temperature of 90 °C to evaporate the solvent. Without any post-treatment, the sample was used as a sensor, after the deposition.

### 2.3. Contacting of the Films

**Figure 2:**
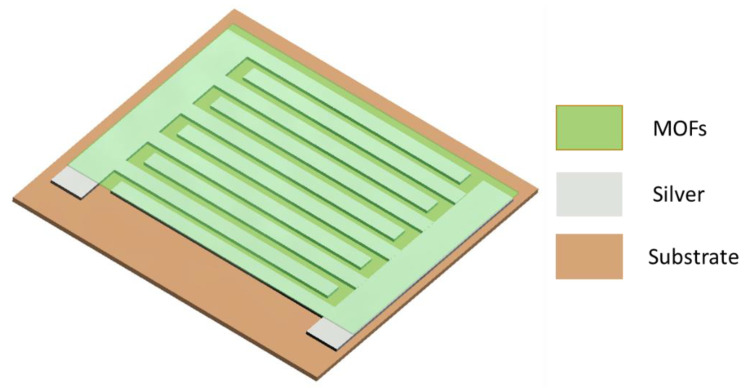
Schematic representation of sensing device.

Electrical contacts were formed to the sensing materials by screen printing highly conductive silver-based (product name: 1010 from Loctite) interdigitated electrode (IDE) structures using a polyester-based mesh with a mesh count of 120 T/cm, as described [21]. The device is based in three layers, a polyimide substrate, silver screen-printed IDE and deposited CP. Over this IDE, the compound is deposited covering all the fingers to create a uniform layer. In Figure 2, the device is shown schematically. The flexibility and conformability area are desired features in many applications, such as wearables and packaging. The conformal substrate employed in this device provides the fabricated sensor with a certain level of both characteristics, expanding the range of applications where such device can be integrated.

The dimensions were selected after printing tests of several combinations of IDE finger widths and spacing (starting from 50 µm to 400 µm). We selected the best compromise of minimal dimensions and high reproducibility. In the case of the selected layout, more than 95% of the fabricated IDEs were fully printed without a short circuit and high resolution.

### 2.4. Single Crystal X-ray Diffraction

Measured crystal was prepared under inert conditions immersed in perfluoropolyether as a protecting oil for manipulation. A suitable crystal was mounted on MiTeGen Micromounts™, and this sample was used for data collection. Data for the compound were collected with a Bruker D8 Venture diffractometer with a photon detector equipped with graphite monochromated *MoKα* radiation (λ = 0.71073 Å). The data were processed with APEX3 suite [22]. The structure was solved by Intrinsic Phasing using the ShelXT program [23], which revealed the position of all non-hydrogen atoms. These atoms were refined on *F*^2^ by a full-matrix least-squares procedure using anisotropic displacement parameters [24]. All hydrogen atoms were located in difference Fourier maps and included as fixed contributions riding on attached atoms with isotropic thermal displacement parameters 1.2 or 1.5 times those of the respective atom. The OLEX2 software was used as a graphical interface [25]. As a consequence of the pseudosymmetries, the least-squares refinements of the structure are not stable, and the use of restraints was required. Some ISOR and RIGU commands had to be used to obtain reasonable displacement parameters for selected non-hydrogen atoms. Crystallographic data for the reported structure have been deposited with the Cambridge Crystallographic Data Center as supplementary publication no. CCDC 2130794. Additional crystal data are shown in Table 1. Copies of the data can be obtained free of charge at http://www.ccdc.cam.ac.uk (accessed on 24 December 2021).

### 2.5. Powder XR Diffraction

**Figure 3:**
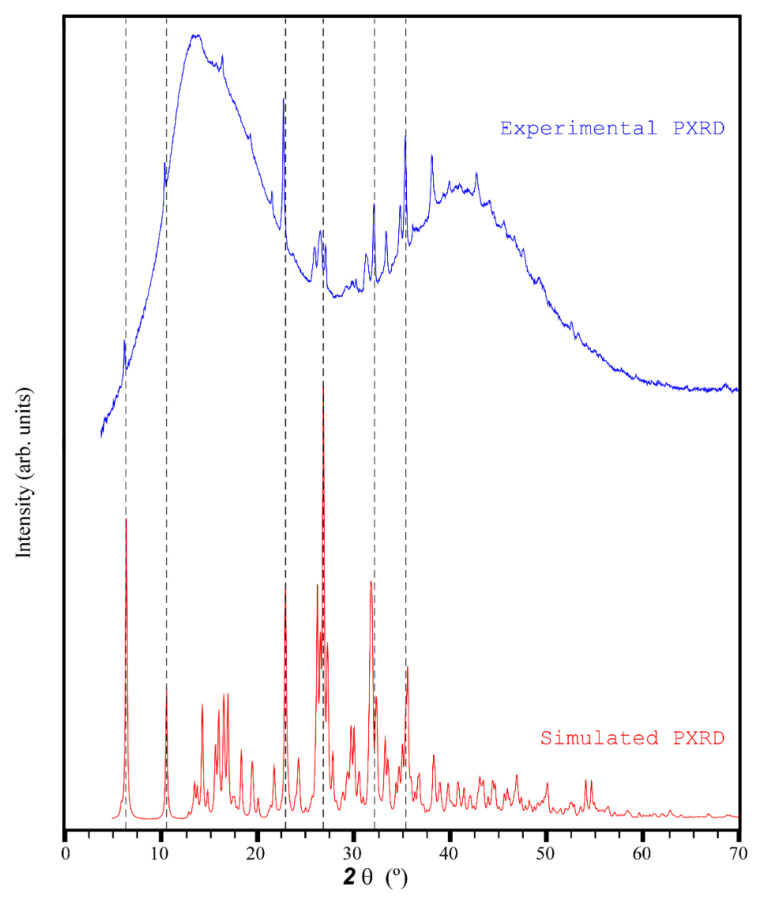
Comparison of the experimental and simulated PXRD patterns of Na-Pery.

The single crystals of Na-Pery were gently ground mixed within oil in an agate mortar and then deposited with care in the hollow of an aluminum holder equipped with a zero-background plate. Diffraction data (Cu Kα, λ = 1.5418 Å) were collected on a *θ*:*θ* Bruker AXS D8 vertical scan diffractometer equipped with primary and secondary Soller slits, a secondary-beam-curved graphite monochromator, a Na(Tl)I scintillation detector, and pulse height amplifier discrimination. The generator was operated at 40 kV and 40 mA. A visual comparison of the experimental and simulated (from single crystal structure) patterns confirms the purity of the bulk. It must be taken into account that only the most intense peaks could be identified due to the strong background signal derived from the oil used in the preparation of the sample, although their positions fit well with those of the simulated pattern (Figure 3).

### 2.6. X-ray Photoelectron Spectroscopy

X-ray photoelectron spectroscopy (XPS) measurements were performed at a base pressure of 5 × 10^−10^ mbar using monochromatic *Kα* radiation from an aluminum anode that is operated at an electrical input power of 350 W. The spectra were acquired using a SPECS (SPECS GmbH) Phoibos hemispherical analyzer at a pass energy of 30 eV with an energy resolution of 0.05 eV. The raw data were processed using the software CasaXPS from Casa Software Ltd. (Teignmouth, UK). The backgrounds of the spectra were removed by Shirley background subtraction [26].

### 2.7. SEM Imaging

Scanning electron microscope (SEM) images were recorded with an NVision40 FESEM from Carl Zeiss (Oberkochen, Germany) at an acceleration voltage of 7 kV, an extraction voltage of 5 kV, and a working distance of 5–6 mm, which was optimized to achieve the best image quality.

### 2.8. Device Characterization

The sheet resistances were measured using a four-point probe head from Jandel (Linslade, UK) connected to a B2901A Keysight (Santa Rosa, CA, USA) source measuring unit (SMU). A constant current of 1 mA was sourced for all measurements. The thicknesses were measured using a DekTak XT profilometer from Bruker (Billerica, MA, USA).

To measure the impedance of the device, a SubMiniature version A (SMA) male connector was glued to electrodes using silver paste. Measurement was carried out with an impedance analyzer (Keysight E4990A). The measurement was conducted at frequencies from 100 Hz to 10 MHz and an amplitude of 500 mV with 0 DC voltage. A calibration was carried out to compensate parasitic elements, as the one performed previously [27]. To automatize the measurements, LabVIEW 2016 was used to control the impedance analyzer.

Temperature and humidity during measurements were controlled in a climatic chamber (VLC4006), and monitorization was conducted with the climatic chamber sensors system. The moisture content was ramped up in 10% steps and kept for 1 h to ensure uniform distribution in the whole chamber volume. All tests were performed 3 times for 2 different fabricated devices.

## 3. Results

### 3.1. Structural Description of the CP

**Figure 4:**
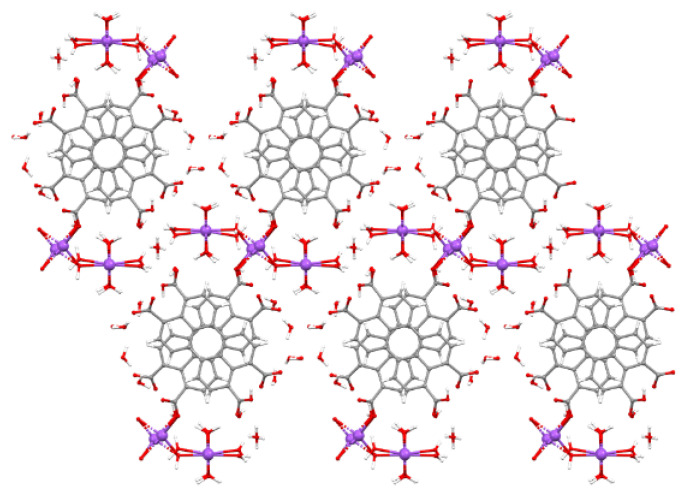
View down the *a* axis of Na-Pery. Color code: H = white, O = red, C = grey, Na = purple.

The conventional reaction of the appropriate amount of 3,4,9,10-perylenetetracarboxylic acid (1 mmol) with NaOH (4 mmol) in water produced prismatic orange crystals of Na-Pery. The crystal structure was determined using single-crystal X-ray diffraction, and its molecular formula is {[Na_5_(ptca)(H_2_O)_18_]·13H_2_O·H_2_ptca}_n_. This compound, unlike the previous published K-Pery MOF [20], crystallizes in the P-1 space group and is described as a 2D-layered structure made of sodium atoms bridged by (ptca)^4−^ linkers and water molecules (Figure 4). In this CP, three different six-coordinated Na^+^ ions are present, all of which possess NaO_6_ environments formed by water and/or carboxylate oxygen atoms. Na1 and Na2 atoms are bridged to one another by some of the water molecules, which act as µ-OH_2_ double bridges, giving rise to infinite chains that run parallel to the *a* axis. On the other hand, one of the water molecules coordinated to Na2 atom that does not take part in the mentioned chain acts as bridge to join to Na3 atoms which, being disordered into two equivalent positions close to an inversion center, complete their hexa-coordination by coordinating to (ptca)^4−^ linkers. Therefore, each (ptca)^4−^ linker bridges two Na3 atoms through carboxylate groups sited on opposite sides of the ligand to render 1D arrays in the [0-1-1] direction, which are, hence, cross-linked with the chains involving Na1 and Na2 atoms to build the 2D layers. Between (ptca)^4-^ linkers, where there is a distance of 6.79 Å, additional protonated ligand (H_2_ptca) molecules are intercalated by means of strong π–π interactions involving the electron clouds of the aromatic rings. Finally, crystallization water molecules occupy the voids of the crystal structure by establishing hydrogen bonding interactions with protonated/deprotonated carboxylate groups and coordinated water molecules.

### 3.2. Characterization of the CP

**Figure 5:**
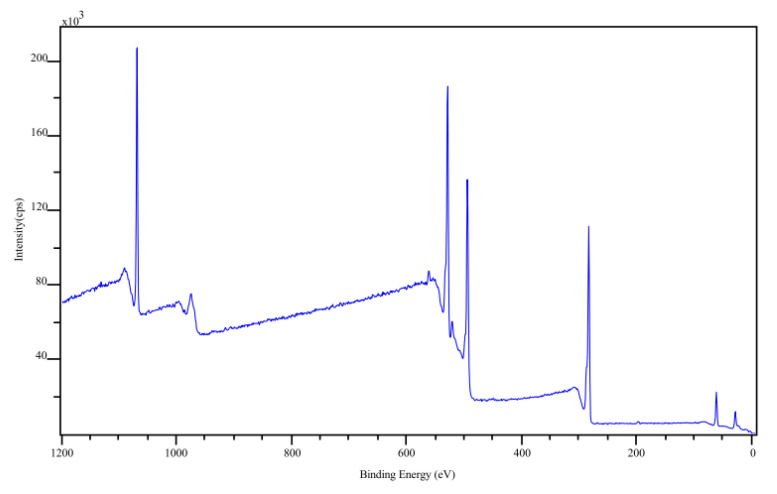
XPS survey scan for Na-Pery sample.

**Figure 6:**
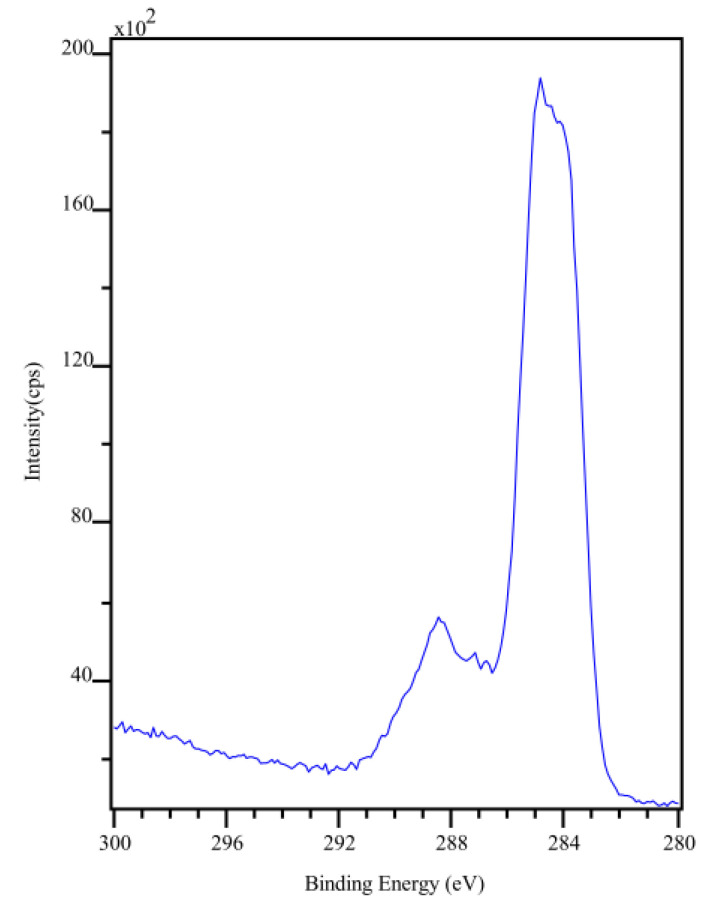
High-resolution XPS scan of the C 1 s transition for (left) Na-Pery. See the text for the contributions at higher-binding energies.

The XPS survey scan and the high resolution XPS scan of the C 1 s transition are shown in Figure 5 and Figure 6 for Na-Pery, respectively. The material was deposited onto polished and doped p-type silicon substrate to prevent charging. From these spectra, it can be seen that Na is present for the Na-Pery sample, which can be recognized by the large peak with a binding energy around 1074 eV [28] that is associated with the Na 1 s transition. Besides this contribution, there is only a negligible amount of carbon associated with the C 1 s transition at a binding energy of 284 eV [29], which is due to contamination and trace residuals by organic solvents. For the C 1 s spectrum, multiple contributions at higher binding energies that are in accordance with the structural formula shown in Figure 1 can be seen in the high-resolution scans shown in Figure 5. The C 1 s spectra are composed of C-C sp² hybridized carbon bonds (284.6 eV), C-C sp³ hybridized carbon bonds (285.6 eV), the carbon-oxygen compounds C-O (286.6 eV), O-C-O (287.6 eV) and O-C=O (289 eV) as well as $\pi -\pi^{*}$ transitions (291 eV). The atomic concentrations for Na-Pery were determined from the XPS survey scan and are summarized in Table 1 along with the theoretical atomic concentrations. The experimental and theoretical values are similar. The increased contribution from carbon in the experimental concentration can be ascribed to organic residuals.

**Figure 7:**
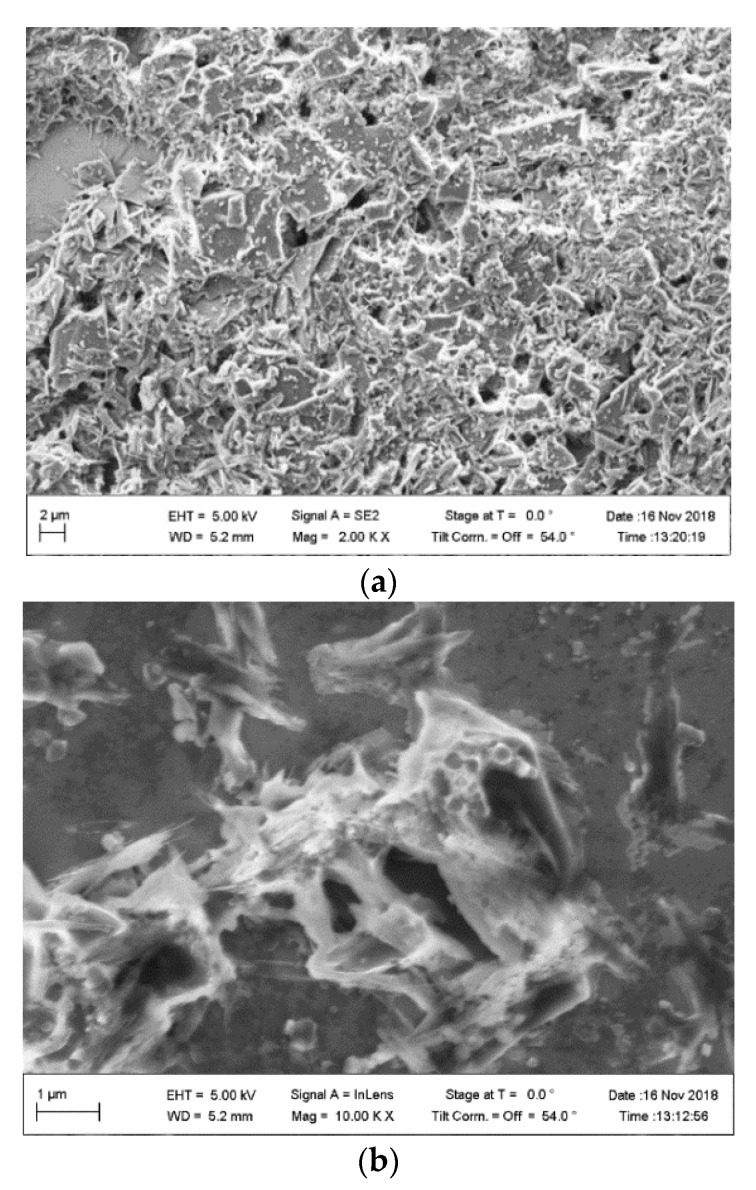
SEM image of Na-Pery (**a**) at 2 kx and (**b**) at 10 kx.

The peak at 534 eV is due to the O 1 s transition and attributed partially to a residual thin water layer that is present on all samples [30]. Besides the water film, there are also contributions from oxygen atoms in the Na-Pery sample. SEM images of the deposited films on the substrate are shown in Figure 7.

**Figure 8:**
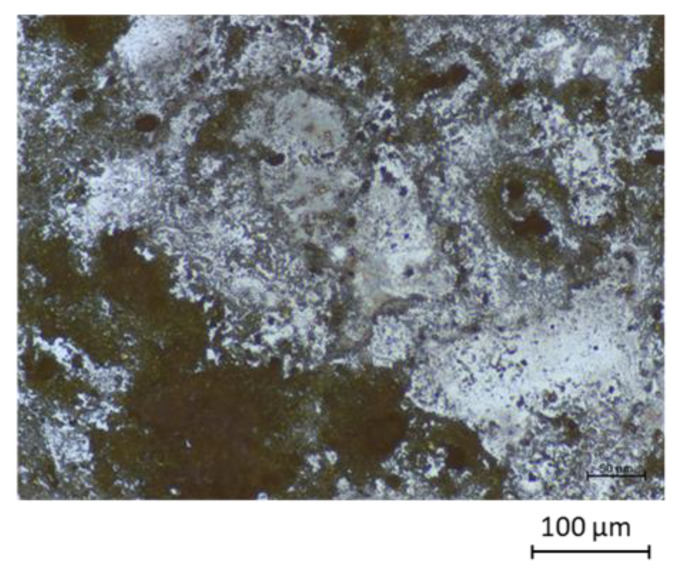
Microscope images of Na-Pery.

In Figure 8, the deposited layers of Na-Pery are shown by using the microscope at a magnification of 20×. In this photograph, it may be observed that the solid possesses low crystallinity in view of the small particle size, which is in agreement with the wide diffraction maxima and the irregular background observed in the diffractogram.

### 3.3. Response to Moisture Content

After printing the electrodes, they showed a thickness of 3.9 µm and a sheet resistance of 79 ± 10 mΩ/sq.

**Figure 9:**
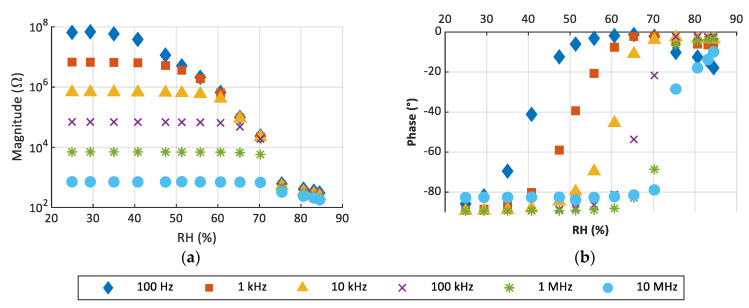
Impedance response towards RH at 40 °C. (**a**) Magnitude and (**b**) phase for Na-Pery drop-casted.

The new CP material was drop-casted to test its response to RH. Given that Na-Pery responded, it was tested with spray deposition. Figure 9 shows the response of the material tested when drop-casted. As can be seen in Figure 9a,b, Na-Pery responds to RH as expected from previous work [20]. In this sense, the response of both Na-Pery and K-Pery below 40% is mainly capacitive, whereas the sensors show a drastic change in the behavior for higher RHs, where the resistive component drops about 5 orders of magnitude, and they become quite conductive. As happened with K-Pery, the response of Na-Pery varies with the frequency excited but always in isolating the state for low RH values and conducting states with higher RH.

**Figure 10:**
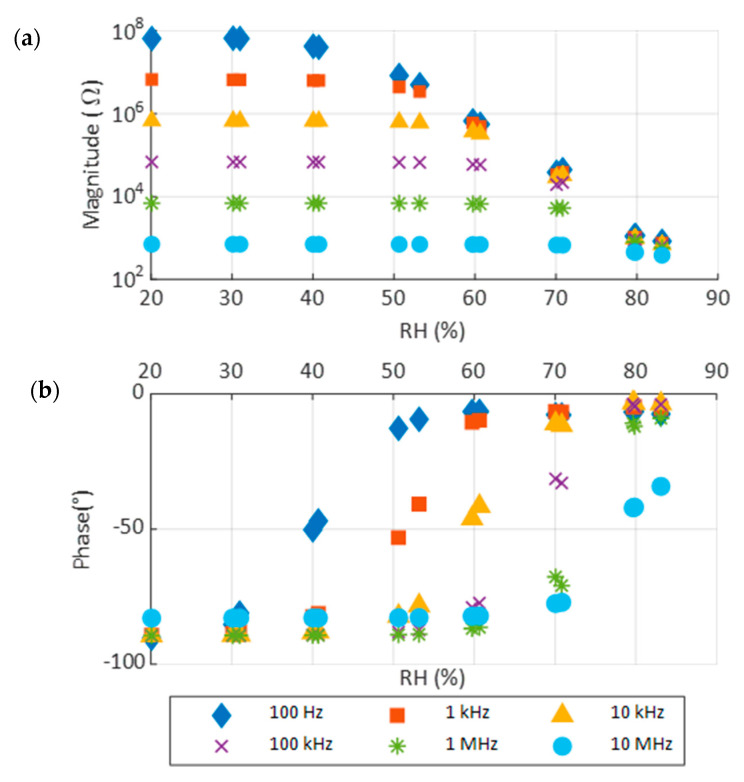
Impedance response towards RH at 40 °C. (**a**) Magnitude and (**b**) phase for spray-deposited Na-Pery.

Spray coating is a more controllable and repeatable system to deposit CP. Na-Pery was tested with spray coating too. In Figure 10, the results for this deposition are shown. As happened with the drop-casted device, a response to RH is observed. However, small differences in this response are appreciated. First, lower conductivity is observed in the case of spray coating as the maximum phase for spray coating is −6.5°, while for drop-casted it is −1.27°. Additionally, the response of the drop-casted device is slightly more abrupt as the high conductive state is reached with less change in RH. These two differences can be explained as a result of the deposition method. With spray coating, the resulted layer has small drops with gapes in between them; this makes it so that when the sensing layer changes its state, less conducting surface is available, so to reach the same state a higher RH level is needed.

The sensitivity of the sensor was studied in the region where the phase changes from capacitive (about −80°) to resistive (about −10°). Actually, outside this region, the sensor response barely varies. The sensitivity at different operating frequencies is shown in Table 2, where the curves were fitted with an exponential model. As can be seen, the sensitivity drastically reduces its value as the frequency increases, achieving a maximum sensitivity of 2.89 × 10^6^ (Ω/%RH) in the range of 29% to 51% at 100 Hz. It should be noted that the range of RH in which the variation can be linearized is reduced as the frequency increases. In addition, the initial value of this range is higher as the frequency increases.

### 3.4. Response to Temperature

**Figure 11:**
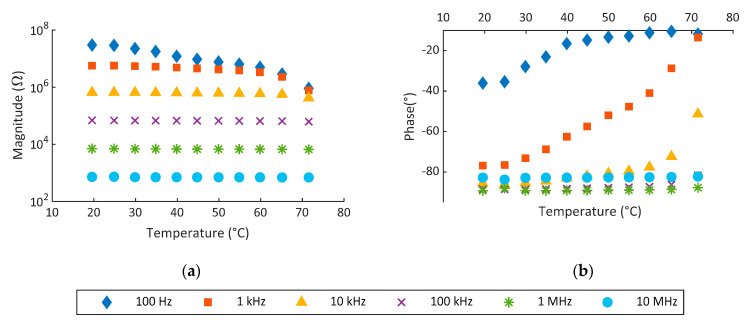
Impedance response towards temperature at RH of 55%. (**a**) Magnitude and (**b**) phase for spray-deposited Na-Pery.

Tests above were carried out at controlled temperature of 40 °C; however, the variation of response with temperature was not analyzed. In Figure 11, the results of these tests are shown. These tests were conducted under an RH controlled condition of 55%. As can be seen, the temperature increases conductivity, especially at lower frequencies. This response is not enough to make the device totally conductive; however, this variation will make the system react at lower RH. At 100 Hz, a saturation effect in phase response is observed for temperatures above 35 °C. Magnitude variation is also higher at lower frequencies; however, it is much lower in comparison to RH response.

## 4. Discussion

The results demonstrate that perylene is not reactive to RH on its own. However, it is possible to functionalize it using different metallic centers. As seen before, perylene combined with sodium reacts to humidity increasing drastically its conductivity when RH is over 40%. The impedance measurements show that the magnitude impedance is reduced up to 5 orders of magnitude and phase changes from −90° to 0°, which is equivalent to pass from a capacitive response to a resistive one, in the case of Na-Pery. This difference in response can be explained due to the different crystalline structure presented by these materials, which directly affects the solubility of alkali metals in water. While in the case of perylene, the structure must present strong stacking interactions between the aromatic rings of the ligand, making their dissolution difficult, in the case of Na-Pery, there are no such interactions, and the coordination to the metal ion generates a trigonal structure with many molecules of water housed in the canals. In this case, sodium is a monovalent ion, similar to the published material of potassium. The sensitivity of the Na-Pery sensor shows higher values when operating at low frequencies, while the range in which the variation is exponential is reduced as the frequency is increased. This behavior of the sensor is consistent with the impedance values: its magnitude is lower with frequency, and the absolute variation in RH is also lower.

In previous work, K-Pery was shown as a RH actuator [20]. In such work, the K-Pery was drop-casted on screen-printed IDEs in an area of about 2 cm^2^. The response is quite similar to the one shown by Na-Pery, reacting around 40% RH and with a resistance in *on* mode around 100 Ω. In terms of temperature, both materials respond, reducing its resistivity, while for Na-Pery, the reduction is higher. Furthermore, in this work, we have made an optimization of the fabrication process by using spray coating and miniaturizing the sensor size. The deposit method employed to deposit the composite does not affect to our results and just modifies the smoothness of the device reaction. This difference is due to the characteristics of the layers created. When using spray coating, Na-Pery keeps in small drops so the electric currents found more resistance to travel from one to another electrode of IDE. Contrarily, when drop-casting, Na-Pery covers the IDE completely; therefore, the current has a bigger amount of material to travel for. However, the use of spray coating is very advantageous in terms of reproducibility and control of the process. Temperature usually affects the humidity sensors. In the case of Na-Pery, the temperature produces a small reaction of the device, increasing its conductivity, especially when the impedance is measured at low frequencies. Despite this reaction, it is much lower than the one by RH. The magnitude is reduced around 1 order of magnitude, which, in comparison to the 5 orders of RH response, is almost negligible.

## 5. Conclusions

In this paper, functionalization of CPs for humidity sensing has been studied. To do so, a CP with Na as metallic centers was created, and its impedance was measured at different RH values. The results show that Na-Pery reacts to humidity when RH is over 40%. These results demonstrate that the center used in the CP is able to modify its sensibility to ambient RH, comparing with the published potassium material. Additionally, the sensibility at temperature changes is modified with the center utilized. Na-Pery has a similar behavior to those with RH but with lower sensibility.

In the case of Na-Pery, the affection of using a different coating method, spray coating, was also analyzed. The study concluded with a similar behavior for drop-casting and spray coating while the spray coating response was less abrupt than the drop-casted sensor.

The use of CPs uncovered in this work can be extremely useful for many applications that need threshold humidity detection. This device can be used in circuits without powering it, which is a great advantage in front of typical RH sensors, which need external devices to read the sensor and check if it crosses the threshold. Furthermore, this device is flexible thanks to the use of conformal substrates, and it can be manufactured with printing techniques. New materials based on alkaline earth ions are being developed in our laboratory to study the effect of the charge of metal ion in modifying the physical properties of these systems.

## Figures and Tables

**Table 1:** Comparison of theoretical and experimentally determined atomic concentrations for Na-Pery.

Element	Atomic Mass	Theoretical Atomic Conc.	Experimental Atomic Conc.
C	12	67%	60%
O	16	22%	28%
Na	22	11%	12%

**Table 2:** Sensitivity of the sensor in the linear response for the different frequencies and range of RH of the linear phase.

Frequency	Sensitivity (Ω/%RH)	Range of RH (%)
100 Hz	−2.89 × 10^6^	29–51
1 kHz	−2.91 × 10^5^	41–61
10 kHz	−3.90 × 10^5^	51–65
100 kHz	−4.38 × 10^3^	61–75
1 MHz	−610	65–75
10 MHz	−501	71–83

## Data Availability

Not applicable.
